# Binding and Retrieval of Response Durations: Subtle Evidence for Episodic Processing of Continuous Movement Features

**DOI:** 10.5334/joc.212

**Published:** 2022-04-07

**Authors:** Roland Pfister, Johanna Bogon, Anna Foerster, Wilfried Kunde, Birte Moeller

**Affiliations:** 1 Department of Psychology, University of Würzburg, Würzburg, DE; 2 Media Informatics Group, Universität Regensburg, Regensburg, DE; 3 Cognitive Psychology, University of Trier, Trier, DE

**Keywords:** Feature binding, stimulus-response binding, metric features, action control

## Abstract

Re-encountering a stimulus retrieves nominally relevant, categorical response features related to previous action decisions in response to this stimulus. Whether binding and retrieval extend to nominally irrelevant, metric features relating to an actual body movement is unknown, however. In two experiments, we thus tested whether repeating target or distractor stimuli across trials retrieves the irrelevant duration of spatial responses to these stimuli. We found subtle indication of such retrieval by task-relevant target stimuli, suggesting that binding and retrieval also operate on metric features of a motor response. In contrast, there was no sign of binding and retrieval of metric features for distractor stimuli. We discuss these observations regarding the representation of action episodes during action-related decision making and during actual movement initiation and control.

## Introduction

Among the major mysteries of human action control is the translation from categorical decisions – say: to open the fridge, or to fetch one’s smartphone – to precise movements. These movements usually involve coordinated activity of different muscles and possibly even multiple effectors, and they are defined by numerous attributes relating to movement trajectories, movement timing, and exerted force. None of these parameters is sufficiently specified by any categorical goal, leaving many degrees of freedom for motor planning and initiation.

The translation from categorical decisions to specific movements thus appears to be a ubiquitous and necessary process for each instance of goal-directed behavior. The interplay of categorical and continuous, metric features of an action has therefore attracted substantial attention in philosophical discourse (e.g., Harleß, 1848; see also Butterfill, & Sinigaglia, 2014; Shepherd, 2019).^1^ Current psychological accounts of action control, by contrast, are surprisingly oblivious to this distinction. This applies especially to recent accounts that highlight feature binding and retrieval as central processes for selecting and initiating actions (Frings et al., 2020; Hommel et al., 2001), and the same holds true for accounts that focus on episodic retrieval across extended timescales (e.g., Logan, 1988).

A key claim of binding and retrieval accounts is that the human cognitive system represents perceptual events and action plans as feature bundles, often labelled event files (Hommel, 2004). These feature bundles result from binding distributed feature codes into integrated representations, e.g., joining color, shape, and location of a visual stimulus to the eventual percept of a unified object (Kahneman et al., 1992). Binding can further incorporate nominally distinct parts of an action episode, including features of the current stimulation, features of a performed action, and features of action-triggered consequences in the environment (Frings et al., 2020). Once bound, any of the features can retrieve the remaining features of the previous episode, so that, e.g., re-encountering a previous situation retrieves a previous action plan.

Research on binding and retrieval in action control has typically employed sophisticated sequential analyses, examining how stimulus repetitions versus switches affect performance for repeated and switched choice responses, respectively. A consistent result of these analyses is that stimulus repetitions promote response repetitions relative to response switches, supporting the idea that features of an action can indeed be bound to (and retrieved by) features of the current stimulation (Hommel, 1998; Frings et al., 2007). But what actually is retrieved in this case? One option is that binding and retrieval of features operate on categorical codes, a suspicion that resonates with the typical categorical design of corresponding experiments. In these experiments, stimuli typically take the form of distinct categories (‘red’ vs. ‘blue’, ‘left’ vs. ‘right’) and so do corresponding responses (typically: ‘left key’ vs. ‘right key’). Some experiments even yielded direct evidence in favor of categorical codes, such as the observation of independent long-term associations between a stimulus and response semantics (e.g., a classification of a picture as showing ‘animals’ vs. ‘objects’) and associations between a stimulus and response identity (e.g., ‘left key’ vs. ‘right key’; Horner & Henson, 2011; Moutsopoulou et al., 2019; Pfeuffer et al., 2018).^2^ Similar evidence comes from the literature on motorvisual priming with an interplay based on categorical features early during movement planning, whereas evidence for metric interactions has mainly been observed after action initiation (Glover & Dixon, 2002; Thomaschke et al., 2012). If binding and retrieval were to operate exclusively on such categorical codes, this would limit the explanatory value of binding and retrieval accounts to capturing the efficiency of decision-making. Only if binding and retrieval (also) operate on continuous, metric properties of a response can these findings be taken to address action control proper.

On closer inspection, the idea of ‘categorical’ motor responses actually is an illusion. There are myriads of ways of carrying out, say, a ‘left’ keypress, differing in where the key is touched, what force is exerted or how long the key is pressed. ‘Left response’ is just a label to summarize this infinite number of motor activities that satisfy an arbitrary criterion set by the experimenter. Given the actual variety of responses with this label, it remains unclear whether and which response properties beyond the arbitrary feature of being ‘left’, are or are not bound to, and retrieved by, stimulus features. The present experiments provide a first direct test of binding and retrieval for the metric feature of response durations.^3^ To this end, we re-analyzed the data of two unpublished experiments that included information on response duration, i.e., the time from response onset to response offset. Assuming that binding and retrieval indeed operate on metric features, we predicted the durations of two successive responses to be more similar in case of stimulus repetitions as compared to stimulus switches, because repeating a stimulus feature from the preceding behavioral episode would trigger retrieval of the previous motor pattern.

## Experiment 1

Assessing binding and retrieval requires experimental setups in which participants respond to successively presented stimuli, and we therefore asked our participants to respond to individual target letters on each trial with two letters mapped to a left response and two letters mapped to a right response, respectively (Frings et al., 2020). Such sequential designs commonly probe for retrieval of previous action plans by analyzing response times and error rates as a function of feature repetitions or switches from a preceding trial (Trial N-1) to the current trial (Trial N). Retrieval can be triggered whenever any feature of the stimulation repeats from Trial N-1 to Trial N, irrespective of whether this feature relates to task-relevant information (operationalized as target stimuli) or task-irrelevant information (operationalized as distractor stimuli; Hommel, 1998; Frings et al., 2007). We therefore aimed at capturing target-response binding and distractor-response binding alike by presenting targets superimposed on distractor stimuli and by varying target and distractor sequences orthogonally across trials.

**Figure 1:**
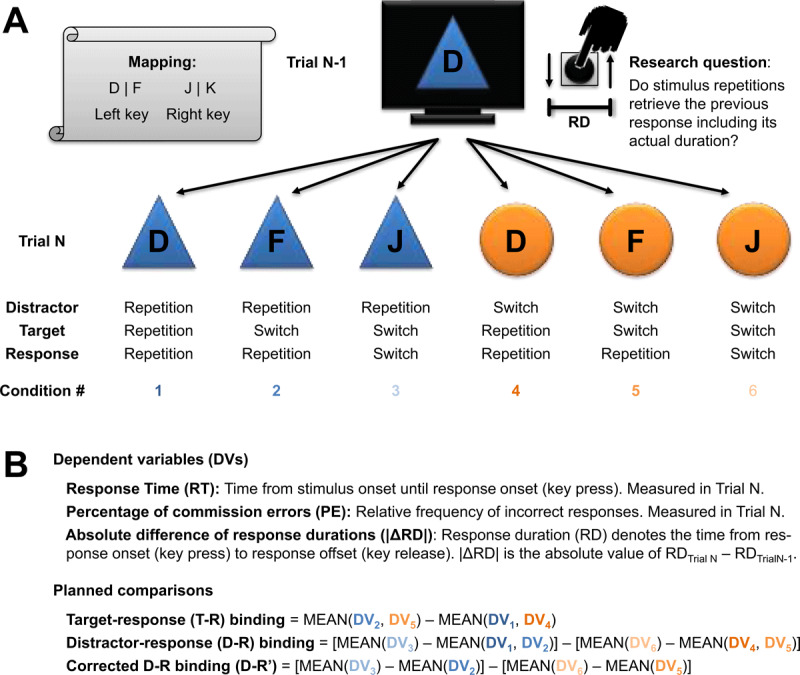
Design and analyses of both experiments. **(A)** Sequential conditions to probe for feature binding and retrieval. The upper display depicts an exemplary preceding trial (Trial N-1) in which the participant had responded to a target letter that was superimposed on a distractor shape. The lower row showcases examples for all possible condition sequences for the participant’s current response (Trial N). **(B)** Dependent variables and planned comparisons. Target-response binding is commonly studied by comparing response repetition trials with target repetitions to response repetition trials with target switches. Distractor-response binding is measured as the difference between response repetitions and response switches for distractor repetition trials relative to distractor change trials. The latter analyses can either include target repetition trials (thus allowing for target-distractor bindings) or exclude target repetition trials for a pure measure of distractor-response binding. Crucially, we applied these analyses to assess whether stimulus repetitions would retrieve the continuous, metric feature of response duration.

***Figure 1*** summarizes all relevant sequential conditions for our main analyses. Target-response binding can be assessed by comparing trials with target repetitions (and thus response repetitions) to trials in which the same response has to be made to a different target as compared to the preceding trial. Faster and more accurate responses in the former condition are suggestive of target-response binding (at least when using relatively simple target stimuli that can be expected not to yield confounding effects of perceptual priming; Pashler & Baylis, 1991). Distractor-response binding, by contrast is measured as the interaction of distractor sequence (repetition vs. switch) and response sequence (repetition vs. switch), i.e., the difference in response repetition benefits between distractor switch trials and distractor repetition trials (see ***Figure 1***).

For the present analyses, target-response binding effects as well as distractor-response binding effects on the performance measures of response times and percentages of commission errors serve as mere manipulation checks. Crucially, following these manipulation checks, we further analyzed the similarity of response durations of successive responses as a continuous, metric property of bindings. We thus computed the absolute difference between response durations (RDs) across successive trials, i.e., |ΔRD| = |RD_current response_ – RD_preceding response_|, and tested this measure with the same analyses as described for response times and error rates.^4^ Observing positive binding effects for |ΔRD| as our main variable of interest would indicate that binding and retrieval does indeed involve continuous, metric features relating to the actual movement. In particular, retrieval of the preceding motor response would bias RD of the current trial to a similar length, diminishing differences between preceding and current RDs.

### Method

#### Apparatus and stimuli

Participants operated a standard German QWERTZ keyboard and were instructed to respond to target letters by pressing either the F key with their left index finger or by pressing the J key with their right index finger. The target set comprised the letters D, F, J, and K, and participants were to respond to the former two letters with a left keypress and they were to respond to the latter two letters with a right keypress. Each target was shown in black font superimposed on a distractor, either an orange circle or a blue triangle. The bounding box of both distractors measured about 2.5 cm × 2.5 cm. Stimulus material and computer code for running the experiments are available on the Open Science Framework (*https://osf.io/8ejax/*).

#### Procedure

Each trial began with a fixation cross for 500 ms followed by target and distractor. This display stayed on screen until the computer registered a keypress. In different experimental blocks, the screen was either blanked upon registering a response (direct offset of target and distractors; responses were followed by a blank screen for 500 ms) or targets and distractors stayed on screen for 500 ms after response onset (delayed offset). During this time, as well as during the fixation display of the next trial, the program sampled additional response events – key presses and key releases – to determine RDs (thus allowing for a maximum RD of 1000 ms).

There were two practice blocks and 14 experimental blocks of 56 trials each. Distractor type (direct offset vs. delayed offset) alternated across blocks, with the initial condition being counterbalanced across participants. Both experiments reported here had originally aimed to determine whether distractor-response binding relates to perceiving the distractor during action planning (perception hypothesis) or whether it actually relates to removing the distractor as a response-contingent event (offset effect hypothesis), as both possibilities can account for the extant literature (see our pre-registration at *https://aspredicted.org/2ty7c.pdf*; for the processing of offset effects, see Gu et al., 2021; Pfister et al., 2012). Results favored the perception hypothesis over the offset effect hypothesis. Due to the striking and consistent results relating to binding and retrieval of RDs, we focus on this intriguing aspect of the results here. We therefore streamlined the results section and report the full analyses, including the factor distractor type, in the Appendix (Tables A1–A3).

#### Participants

We recruited 40 participants. Their mean age was 26.7 years (SD = 10.1; range: 18–66 years); 27 self-identified as female, 13 as male, 35 were right-handed as assessed via self-report. This sample size provides a power of 80% for effect sizes of Cohen’s *d_z_* = 0.45 and a power of 90% for d_z_ = 0.53, and we had intended to have a power of 80% for medium-sized effects after participant exclusions (which would correspond to an effective sample size of at least 34 participants). Even though binding and retrieval effects are often large in the literature (e.g., d_z_ = 1.15 for Exp. 1a in Frings et al., 2007), we opted for such conservative effect size estimates because our main comparisons of interest had not been tested so far, especially not concerning any effects on the dependent variable of |ΔRD|. As per our pre-registration, two participants had to be excluded due to an error rate of more than 20% in at least one design cell.

### Results

**Figure 2:**
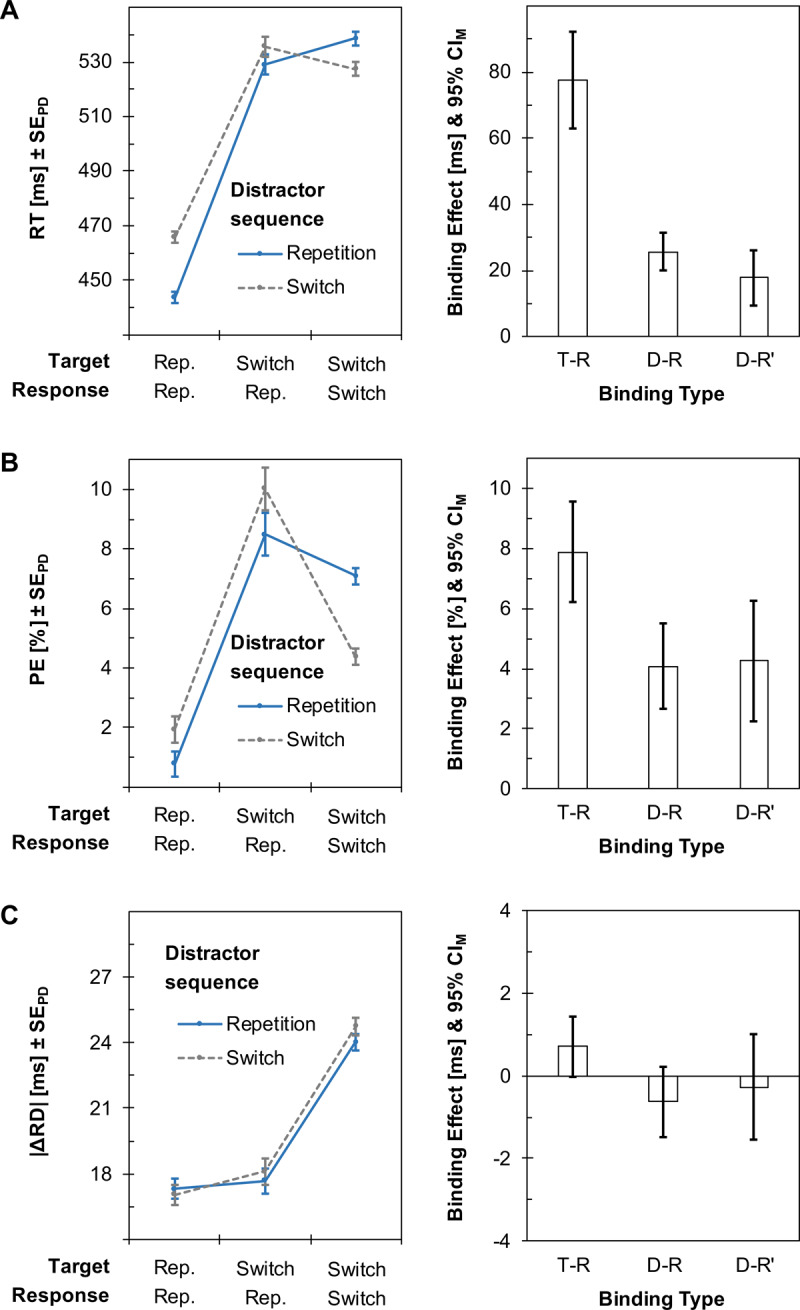
Main results of Experiment 1. Left plots summarize mean response times (RTs; Panel A), percentages of commission errors (PEs; Panel B), and absolute differences in response durations between successive trials (|ΔRD|; Panel C). Error bars show standard errors of paired differences (SE_PD_). All variables were analyzed as a function of distractor sequence as well as target sequence and response sequence. Right plots show corresponding binding effects between targets and responses (T-R) as well as between distractors and responses, computed either on the full dataset (D-R) or on a reduced dataset after excluding target repetition trials (D-R’). Error bars show 95% confidence intervals of the individual means (CI_M_).

***Figure 2*** summarizes the critical findings of the experiment. This especially comprises the difference scores to capture target-response binding and distractor-response binding. Binding and retrieval effects were computed as described in the introduction (see also ***Figure 1***), and we will refer to these effects as Δ when reporting the results.

Raw data and analysis scripts are available on the Open Science Framework (*https://osf.io/8ejax/*). Due to the sequential nature of the analyses, we excluded the first trial of each block, as this trial does not have an immediate predecessor, and we also did not analyze trials following an error to avoid potential effects of post-error slowing (e.g., Pfister & Foerster, 2021). For all analyses of response times and |ΔRD| we further removed trials for which either measure deviated more than 2.5 standard deviations from the corresponding cell mean (5.0% of the data).

#### Manipulation checks

Response times showed a large effect of target-response binding, *t*(37) = 10.82, *p* < .001, Δ[binding effect] = 78 ms, 95% CI = [63 ms, 92 ms], *d_z_* = 1.75, 95% CI_d_ = [1.24, 2.26] (confidence intervals for standardized effect sizes, denoted as “CI_d_”, were computed with the MBESS package in R, whereas confidence intervals for the raw differences scores were computed as standard error × quantile of the *t* distribution and are denoted as “CI” without index.) There was also large distractor-response binding when computed on the full dataset, *t*(37) = 9.00, *p* < .001, Δ = 26 ms, 95% CI = [20 ms, 31 ms], *d_z_* = 1.46, 95% CI_d_ = [1.00, 1.91], whereas a medium-sized effect remained after removing target repetitions from the dataset, *t*(37) = 4.33, *p* < .001, Δ = 18 ms, 95% CI = [9 ms, 26 ms], *d_z_* = 0.70, 95% CI_d_ = [0.34, 1.05] (see ***Figure 1*** for examples of each sequential condition and the computation of each binding effect).

Similarly, error rates yielded a large effect of target-response binding, *t*(37) = 9.63, *p* < .001, Δ = 7.89%, 95% CI = [6.23%, 9.55%], *d_z_* = 1.56, 95% CI_d_ = [1.08, 2.03], and distractor-response binding when using the entire dataset, *t*(37) = 5.78, *p* < .001, Δ = 4.07%, 95% CI = [2.64%, 5.50%], *d_z_* = 0.94, 95% CI_d_ = [0.55, 1.32]. Distractor-response binding again amounted to a medium-sized effect for the reduced dataset after removing target repetitions, *t*(37) = 4.28, *p* < .001, Δ = 4.25%, 95% CI = [2.24%, 6.27%], *d_z_* = 0.69, 95% CI_d_ = [0.34, 1.05].

#### Response durations

The key measure |ΔRD| yielded a small descriptive difference between target repetitions and target switches though target-response binding did not reach the conventional level of significance, *t*(37) = 1.98, *p* = .055, Δ = 0.71 ms, 95% CI = [–0.02 ms, 1.43 ms], *d_z_* = 0.32, 95% CI_d_ = [–0.01, 0.64].

There were no signs of distractor-response binding for |ΔRD|, neither as computed on the entire dataset, *t*(37) = 1.49, *p* = .132, Δ = –0.63 ms, 95% CI = [–1.48 ms, 0.22 ms], *d_z_* = 0.–22, 95% CI_d_ = [–0.54, 0.10], nor after removing target repetitions from the data, *t*(37) = 0.43, *p* = .668, Δ = –0.27 ms, 95% CI = [–1.55 ms, 1.01 ms], *d_z_* = –0.07, 95% CI_d_ = [–0.39, 0.25].

### Discussion

The results yielded subtle evidence for the hypothesis that continuous, metric properties of an action such as its duration becomes bound and later retrieved upon re-encountering its accompanying stimuli, whereas there were no signs whatsoever for distractor-response binding. Before drawing meaningful conclusions from this pattern, we aimed at replicating the analysis on a second, comparable dataset.

## Experiment 2

For the present purposes, Experiment 2 presents a close replication of Experiment 1 with minor methodological adjustments as described below. We expected to replicate target-response binding effects as well as distractor-response binding effects in response times and percentages of commission errors, pointing to binding of categorical action features and second, and asked whether binding and retrieval for |RDs| would support the notion of binding for continuous action features.

### Method

#### Apparatus, stimuli, and procedure

The experiment was a direct replication of Experiment 1 with the sole exception that targets were now de-coupled from distractors in half of the blocks. That is: Targets always disappeared with response onset whereas distractors either disappeared directly or only after a delay of 500 ms. We again focus on the most critical results in the main text, collapsed across distractor types, because the results did not depend on target-distractor timing. We further replicated the findings in the overall analyses by specific analyses of de-coupled blocks in case of binding and retrieval of RDs. As for Experiment 1, the full analyses including all design factors are described in the Appendix.

#### Participants

We recruited 45 participants. The sample size considerations followed the same rationale as for Experiment 1 though we collected five additional participants for organizational reasons. The participants’ mean age was 25.0 years (SD = 6.3; range: 19–51 years); 40 self-identified as female, 5 as male, 40 were right-handed, and 5 were left-handed as assessed via self-reports. Four participants were excluded based on the same criterion as in Experiment 1, i.e., because error rates exceeded 20% in at least one design cell.

### Results

**Figure 3:**
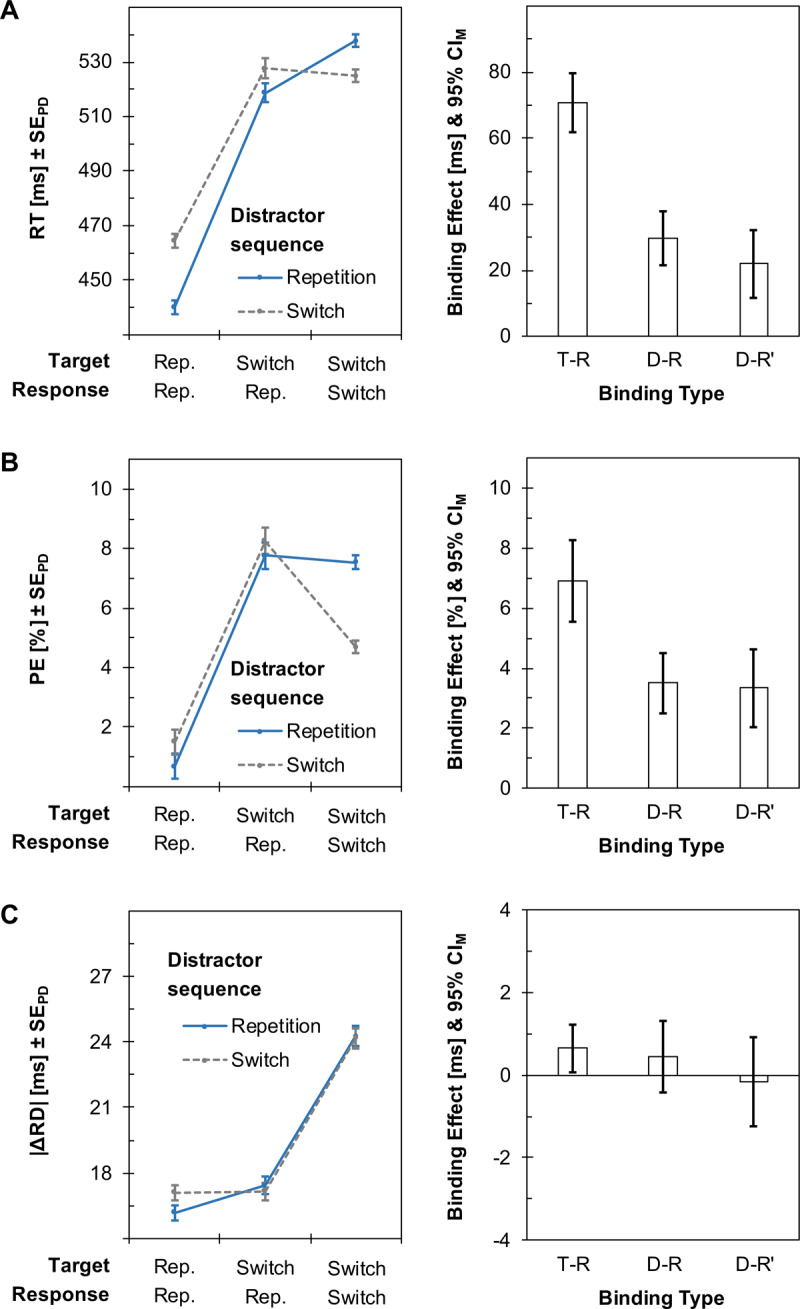
Main results of Experiment 2. Left plots summarize mean response times (RTs; Panel A), percentages of commission errors (PEs; Panel B), and absolute differences in response durations between successive trials (|ΔRD|; Panel C). Error bars show standard errors of paired differences (SE_PD_). All variables were analyzed as a function of distractor sequence, as well as target and response sequence. Right plots show corresponding binding effects between targets and responses (T-R) as well as between distractors and responses, computed either on the full dataset (D-R) or on a reduced dataset after excluding target repetition trials (D-R’). Error bars show 95% confidence intervals of the individual means (CI_M_).

***Figure 3*** summarizes the critical findings of the experiment (see Tables A4–A6 in the Appendix for full analyses). Analyses were as for Experiment 1, and we excluded 4.94% of the trials as outliers for analyses of response times and |ΔRD|. Raw data and analysis scripts are available online (*https://osf.io/8ejax/*).

#### Manipulation checks

Response times showed a large effect of target-response binding, *t*(40) = 15.79, *p* < .001, Δ = 71 ms, 95% CI = [62 ms, 88 ms], *d_z_* = 2.47, 95% CI_d_ = [1.84, 3.08]. A large effect also emerged for distractor-response binding when computing it on the full dataset, *t*(40) = 7.25, *p* < .001, Δ = 30 ms, 95% CI = [21 ms, 38 ms], *d_z_* = 1.13, 95% CI_d_ = [0.73, 1.52], with a medium-sized effect after removing target repetitions from the dataset, *t*(40) = 4.23, *p* < .001, Δ = 22 ms, 95% CI = [12 ms, 32 ms], *d_z_* = 0.68, 95% CI_d_ = [0.33, 1.01].

Error percentages also yielded a large effect of target-response binding, *t*(40) = 10.34, *p* < .001, Δ = 6.91%, 95% CI = [5.56%, 8.27%], *d_z_* = 1.62, 95% CI_d_ = [1.14, 2.08], and of distractor-response binding when using the entire dataset, *t*(40) = 7.09, *p* < .001, Δ = 3.51%, 95% CI = [2.51%, 4.51%], *d_z_* = 1.11, 95% CI_d_ = [0.71, 1.49]. A large effect of distractor-response binding was also evident for the reduced dataset after removing target repetitions, *t*(40) = 5.21, *p* < .001, Δ = 3.35%, 95% CI = [2.05%, 4.64%], *d_z_* = 0.81, 95% CI_d_ = [0.46, 1.16].

#### Response durations

The key measure |ΔRD| again only yielded subtle signs of target-response binding, *t*(40) = 2.26, *p* = .029, Δ = 0.65 ms, 95% CI = [0.07 ms, 1.24 ms], *d_z_* = 0.35, 95% CI_d_ = [0.04, 0.67]. As in Experiment 1, there were no signs of distractor-response binding, neither as computed on the entire dataset, *t*(40) = 1.02, *p* = .316, Δ = 0.44 ms, 95% CI = [–0.43 ms, 1.31 ms], *d_z_* = 0.16, 95% CI_d_ = [–0.15, 0.47], nor after removing target repetitions from the data, *t*(40) = 0.32, *p* = .752, Δ = –0.17 ms, 95% CI = [–1.24 ms, 0.91 ms], *d_z_* = –0.05, 95% CI_d_ = [–0.36, 0.26].

### Discussion

Experiment 2 replicated the small binding effect observed for Experiment 1: RDs were more similar for trials with repetitions of task-relevant stimuli alike, suggesting that continuous, metric features such as RDs do indeed become bound and can be retrieved later on. This effect was again substantially smaller than the effects observed for response times and error rates, and there was no evidence for binding and retrieval of RDs by irrelevant distractor stimuli.

## Pooled analyses

A final analysis targeted the pooled data of both experiments to arrive at a credible estimate of a possible effect size for target-response binding for RDs. Here, target-response binding and retrieval amounted to an effect size of *d_z_* = 0.34 for the pooled |ΔRD| data, 95% CI_d_ = [0.11, 0.56]. We did not analyze distractor-response binding further because both individual experiments had yielded clear evidence against such an effect.

## General Discussion

The present experiments aimed at studying whether the continuous feature of RD becomes bound to, and is retrieved by, stimuli that precede the response. RDs are a particularly subtle property of each action in the present experimental design, because participants only decided between spatially arranged keypress responses whereas the eventual durations did not play any role for the task at hand. Unlike in previous studies in which RD was a task-relevant response features (e.g. Kunde & Stöcker, 2002) it was thus task-irrelevant here. The distinction of relevant (spatial) and irrelevant (temporal) response features thus parallels a common distinction between nominally relevant aspects of stimulation (targets) and nominally irrelevant stimulation (distractors).

The results revealed large and robust evidence for binding between nominally relevant response features with nominally relevant stimulus features as well as with nominally irrelevant stimulus features. For nominally irrelevant RDs, by contrast, there was no evidence of binding and retrieval with nominally irrelevant distractor features whereas subtle evidence pointed to binding and retrieval with nominally relevant target features as measured in absolute differences between durations of successive responses.^5^ This pattern seems to suggest that the combined task relevance of two features influences binding and retrieval between these features (for a similar argument, see Moeller et al., 2019). Task-relevant response features bind to and retrieve relevant and irrelevant stimulus features alike, while for irrelevant response features, the stimulus feature has to be task-relevant for binding effects to occur. Corresponding effects came with a small effect size, however (actually the numerically smallest effects we have ever observed in experiments on binding and retrieval when measured in milliseconds rather than standardized effect size). Yet, they consistently occurred in two experiments and were significant in a high-powered, pooled analysis. We therefore feel uncomfortable with simply neglecting these small effects, especially because the dependent measure indexing these effects, |ΔRD|, came with a very low baseline level anyway (about 20 ms). The scale of the observed effects on |ΔRD| therefore cannot be compared to common values for response time analyses, and target-response binding amounted to 3.6% and 3.4% of the overall |ΔRD| range for Experiment 1 and 2, respectively. The results therefore seem to suggest partial retrieval, likely relating to temporal aspects of kinesthetic and proprioceptive action effects (Pfister, 2019), and they thus indicate recycling of the motor plan underlying a previous action. Keeping these caveats in mind, we therefore want to discuss the theoretical implications of these effects.

The observation of binding and retrieval effects for the continuous, metric feature of RD supports the notion that these processes operate not only during action-related decision making, but they also seem to comprise features of the ensuing body movement instead. This conclusion therefore partly backs current theoretical accounts of binding and retrieval as capturing crucial mechanisms of action selection and planning (Frings et al., 2020). At the same time, the present results suggest that movement-related features only play a subordinate role for binding and retrieval. Yet, we need to keep in mind that movement-related features (but not categorical features) were task irrelevant in the present study and binding and retrieval effects may well increase with increasing relevance of the features involved.

Accepting that binding and retrieval might also capture continuous features of actual body movements begs the question of whether action-related decision making on the one hand and action planning on the other hand reflect a gradual evolution of a single process or whether they are largely independent instead. Effect-based accounts suggest that the continued activation of (the same) features underlies selection, planning, and initiation of an action (Kunde et al., 2004). Other approaches, however, have explored a range of different scenarios, including those that assume a clear separation of decision making and action (e.g., Bernier et al., 2012; for an overview and classification of different accounts, see Lepora & Pezzulo, 2015).

Even though the present results do not speak directly to the question of separation versus integration of decision making and action, several aspects of the data appear to be more compatible with a separate view. For one, effects on response times and |ΔRD| were statistically independent even for the pooled analysis, which provided sufficient power for medium-sized correlations of at least *r* = .30. For another, RDs were always highly similar for repeated responses, irrespective of whether these responses were made to a repeated target or in response to a different target stimulus. Switching the response, however, resulted in markedly dissimilar RDs relative to both conditions with repeated responses. The pattern for response times and error rates differed from this observation in that the stimulus sequence played a much more pronounced role than the sequence of responses. This pattern would follow seamlessly from a system that compiles an action decision first and then involves additional processes to enact this decision. Note that the residual effects for target-response binding and retrieval also do not necessarily stem directly from perceiving a previously encountered target. Instead, response decisions involving the relevant spatial feature of a repeated response might retrieve the previous RD at least to some extent. Future research is required to address this question.

Assuming distinct features to predominate during decision making and action planning also resonates with recent observations on binding and retrieval after action slips (Foerster et al, 2021a, b). Here, re-encountering a stimulus that had been present at the time of error commission reliably retrieved the intended correct response rather than the erroneous response that had actually been executed. Re-encountering the effect of an erroneous action, by contrast, retrieved the actually performed action. Moreover, corresponding binding and retrieval effects of relevant stimuli after commission errors were smaller than those after correct responses. Because this difference could not be explained by factors such as post-error slowing or by assuming counteracting effects of retrieving either the erroneous or the correct response across different trials, this pattern likely suggests that binding can leverage richer and more features in case of an actually executed response. This view thus seems to suggest that action planning and execution partly relies on distinct features as compared to action-related decision making (for conceptually related observations on the role of action execution in the field of task switching, see Philipp et al., 2007; Schuch & Koch, 2003). Interestingly, the described experiments on binding for action slips did not reveal any binding and retrieval effects for RDs, neither following erroneous responses nor following correct responses. Absent binding and retrieval effects for movement-related features would be expected for goal-based binding after erroneous responses following the above argument. Not observing such effects for correct responses, however, suggests that such binding and retrieval effects depend on contextual factors. One potential factor might be response caution, as the discussed work on binding for action slips (Foerster et al., 2021a, b) used sharper response deadlines than the present setup, thus likely shifting decision criteria towards speed rather than towards accuracy. Future work should thus aim at delineating when binding and retrieval apply exclusively to categorical, decision-related features and when they also apply to continuous, movement-related features.

The current metric measure of RD for simple keypresses further comes with relatively limited variance. Durations of more extended movements and other metric characteristics such as spatial movement trajectories likely yield larger variance and might thus be affected more easily by experimental manipulations (for findings of feature-based interference between concurrently active plans for different actions, see Fournier et al., 2015; Wiediger & Fournier, 2008).^6^ It also remains to be tested how episodic processing of RD changes when that duration becomes a relevant aspect of the response. Preliminary evidence suggests that planning a response with a certain categorical duration (short or long) shifts the actual duration of another response in the opposite direction (i.e., keeping in mind a short response results in longer RDs of intermittently requested actions; Mocke, et al., in press). Probing such additional parameters may thus yield converging evidence for the present speculations. Albeit RD varied to a limited extent in the present study, this duration must still have been encoded in the episodic representation of the response in order to be retrieved later. Under which conditions such encoding occurs, which amount of attention such encoding requires and so on, requires more research.

Because movements are defined by a rich and diverse set of features, a compelling research agenda would ideally aim to include novel measures relating to a movement’s spatial properties and particularly the force that is exerted during the movement (see Varga et al., submitted to this Special Issue). Understanding when and how such parameters are bound and retrieved will provide the grounds for connecting research on action control with research on motor control proper. It will thus address how efficiently binding and retrieval work towards the eventual goal of action control mechanisms, that is: building a bridge from cognition to movement.

## Data Accessibility Statement

Stimulus material, computer programs, raw data, and analysis scripts are available on the Open Science Framework (*https://osf.io/8ejax/*; doi: *10.17605/OSF.IO/8EJAX*).

## Footnotes

A related philosophical distinction concerns conceptual versus non-conceptual content of perceptual (Evans, 1982) and action representations (Pacherie, 2011).

The same argument also applies to stimulus codes that can either be metric representations of a sensory signal (say: the exact hue of a perceived color) or categorical abstractions (e.g., Frings et al., 2013; Pfeuffer et al., 2018). This latter option blurs the operational distinction between stimuli and responses by highlighting the possibility of incorporating classification responses rather than actual stimulus representations into episodic binding (for recent evidence on response-response binding, see Moeller & Frings, 2019).

Binding and retrieval of categorical duration features in terms of ‘short’ versus ‘long’ stimulus durations has previously been reported in the literature (Bogon et al., 2017). These findings might be taken to suggest that temporal features can be bound and retrieved. However, as with categorical spatial features (e.g., ‘left’), observing binding and retrieval for such response categories would relate to (categorical) decision making rather than action planning and initiation. Studies on human action control that addressed RDs as metric properties are few and far between (Cox & Hasselmann, 2013; Foerster et al., in press; Horváth et al., 2018; Kunde, 2003), and we are not aware of any such studies in the context of binding and retrieval.

Note that we opted to analyze |ΔRD| rather than mean RD because RDs on their own are not particularly informative for binding and retrieval. Whereas common measures such as response times or error rates come with a direct interpretation – short response times and low error rates indicate that action planning and/or action initiation were efficient and easy – this is not necessarily the case for RDs. In a binding and retrieval context, short response times and low error rates for stimulus repetitions can therefore be taken to indicate that a previous response to the same stimulus was retrieved. Retrieving a response, however, should not necessarily lead to shorter RDs. To the contrary: Retrieving the actual motor action should cause the whole response to be similar to the preceding one, including parameters such as duration, force profile etc. This is why we chose to analyze |ΔRD| as an index of similarity. If this number is small then the durations of the current response and of the preceding response are similar, whereas large values indicate dissimilarity. An alternative way to quantify similarity would be to assess intra-individual correlations of successive RDs as explored by a recent, conceptually related study (Varga et al., submitted to this Special Issue). Using this alternative method fully replicated the pattern of results for the present dataset.

Despite the negative results of the present experiments, distractor-response binding for response durations or other continuous features of a response might still emerge for alternative experimental setups. Promising candidates include setups with auditory distractors because such stimuli are difficult to ignore (e.g., Moeller et al., 2016).

Despite their limited variance, response durations appear to be an overlooked and easily accessible parameter for any experiment that assesses response times. Not only do they seem to be uncorrelated with response time effects but they can even showcase unique patterns, e.g., during error processing (Foerster et al., in press).

## Appendix

Table A1 shows mean response times, percentages of commission errors, and absolute differences in RDs between two successive trials (|ΔRDs|) for all design cells of Experiment 1. Table A2 summarizes inferential results for the corresponding omnibus analyses of variance (ANOVAs) for each measure, and Table A3 shows the individual binding effects, separately for the direct offset condition and the delayed offset condition. Table A4 shows descriptive statistics for Experiment 2. Table A5 summarizes inferential results for the corresponding omnibus ANOVAs, and Table A6 shows the individual binding effects.

Even though the exact effect sizes for binding varied across experiments, conditions, and dependent variables, there was at least one binding and retrieval effect of medium to large size for each distractor type, i.e., for direct offsets and delayed offsets alike. We also did not observe any systematic differences in terms of binding and retrieval effects between direct offsets and delayed offsets. We had originally speculated that delayed offsets might yield smaller effects than the standard setting of immediate post-response offsets of targets and distractors, because targets and distractors can be conceptualized as offset effects of the preceding response in addition to their role of being present at the time of action planning and initiation (see the preregistration at *https://aspredicted.org/2ty7c.pdf*). This prediction does not seem to align with empirical reality, however. The results therefore suggest that distractor-response binding solely derives from the perception of the distractor at the time of action planning and initiation.

**Table A1:** Mean response times (RTs [ms]), percentages of commission errors (PEs [%]), and absolute differences in response durations across successive trials (|ΔRD| [ms]) as a function of all three design factors of Experiment 1.

		DIRECT OFFSET			DELAYED OFFSET		

		TARGET REPETITION	TARGET SWITCH	TARGET SWITCH	TARGET REPETITION	TARGET SWITCH	TARGET SWITCH

DV	DISTRACTOR SEQUENCE	RESPONSE REPETITION	RESPONSE REPETITION	RESPONSE SWITCH	RESPONSE REPETITION	RESPONSE REPETITION	RESPONSE SWITCH

RT	Repetition	438.99	510.13	528.89	448.28	547.91	548.55

	Switch	456.86	524.76	518.60	474.58	546.35	536.27

PE	Repetition	0.93	10.17	7.68	0.63	6.81	6.51

	Switch	2.10	11.02	4.69	1.78	9.02	4.06

|ΔRD|	Repetition	16.87	17.47	24.43	17.77	17.87	23.64

	Switch	17.03	18.15	25.16	17.04	18.05	24.33

**Table A2:** Omnibus analyses of variances (ANOVAs) for each dependent variable (DV) of Experiment 1. Mean response times (RTs), percentages of commission errors (PEs), and absolute differences in response durations across successive trials (|ΔRD|) as a function of all three design factors of Experiment 1. The factors distractor type (direct offset vs. delayed offset) and distractor sequence (repetition vs. switch) come with two levels each, whereas the factor response sequence codes the three levels of all possible target and response sequences (i.e., target repetition with response repetition, target switch with response repetition, and target switch with response switch). ANOVA effects for which the sphericity assumption was violated were corrected by the Greenhouse-Geisser method, and we provide the corresponding ɛ estimate for each of these tests.

DV	SOURCE	F	P	η_P_^2^	ɛ

RT	Distractor Type (DT)	43.50	<.001	0.54	

	Distractor Sequence (DS)	13.54	<.001	0.27	

	Response Sequence (RS)	109.51	<.001	0.75	.761

	DT * DS	0.88	.355	0.02	

	DT * RS	5.71	.005	0.13	

	DS * RS	34.15	<.001	0.48	.853

	DT * DS * RS	3.99	.039	0.10	.688

PE	Distractor Type (DT)	9.32	.004	0.20	

	Distractor Sequence (DS)	0.00	.973	0.00	

	Response Sequence (RS)	71.79	<.001	0.66	.754

	DT * DS	1.09	.303	0.03	

	DT * RS	4.62	.021	0.11	.786

	DS * RS	18.81	<.001	0.34	.660

	DT * DS * RS	0.39	.641	0.01	.841

|ΔRD|	Distractor Type (DT)	0.07	.797	0.00	

	Distractor Sequence (DS)	0.91	.346	0.02	

	Response Sequence (RS)	49.44	<.001	0.57	.606

	DT * DS	0.94	.337	0.02	

	DT * RS	2.02	.140	0.05	

	DS * RS	1.23	.294	0.03	.813

	DT * DS * RS	0.26	.770	0.01	

**Table A3:** Binding and retrieval effects for response times (RT [ms]), percentages of commission errors (PE [%]), and absolute differences in response durations across successive trials (|ΔRD| [ms]) for Experiment 1. Separate binding and retrieval effects were computed for targets and responses (T-R) as well as for distractors and responses, with the latter being computed on the entire dataset first (D-R) and also on a reduced dataset after excluding target repetition trials (D-R’).

		DIRECT OFFSET			DELAYED OFFSET		

DV	STATISTIC	T-R	D-R	D-R’	T-R	D-R	D-R’

RT	Mean	69.83	26.54	24.92	85.39	24.66	10.73

	SE_M_	6.44	3.52	4.58	8.51	4.61	7.86

	d_z_	1.76	1.22	0.88	1.63	0.87	0.22

PE	Mean	8.08	4.00	3.84	7.70	4.14	4.67

	SE_M_	0.92	1.11	1.50	0.87	0.87	1.20

	d_z_	1.42	0.59	0.42	1.44	0.77	0.63

|ΔRD|	Mean	1.07	–0.30	–0.04	0.35	–0.96	–0.50

	SE_M_	0.38	0.61	0.94	0.65	0.58	0.72

	d_z_	0.46	–0.08	–0.01	0.09	–0.27	–0.11

**Table A4:** Mean response times (RTs [ms]), percentages of commission errors (PEs [%]), and absolute differences in response durations across successive trials (|ΔRD| [ms]) as a function of all three design factors of Experiment 2.

		DIRECT OFFSET			DELAYED OFFSET		

		TARGET REPETITION	TARGET SWITCH	TARGET SWITCH	TARGET REPETITION	TARGET SWITCH	TARGET SWITCH

DV	DISTRACTOR SEQUENCE	RESPONSE REPETITION	RESPONSE REPETITION	RESPONSE SWITCH	RESPONSE REPETITION	RESPONSE REPETITION	RESPONSE SWITCH

RT	Repetition	439.53	514.97	535.48	441.01	522.49	540.21

	Switch	462.35	522.17	520.92	466.88	533.12	528.70

PE	Repetition	0.98	7.56	7.69	0.38	7.96	7.39

	Switch	1.70	8.75	4.89	1.32	7.77	4.50

|ΔRD|	Repetition	16.21	17.50	24.65	16.13	17.36	23.90

	Switch	17.03	16.93	24.13	17.20	17.38	24.20

**Table A5:** Omnibus analyses of variances (ANOVAs) for each dependent variable (DV) of Experiment 2. Mean response times (RTs), percentages of commission errors (PEs), and absolute differences in response durations across successive trials (|ΔRD|) as a function of all three design factors of Experiment 2. The factors distractor type (direct offset vs. delayed offset) and distractor sequence (repetition vs. switch) come with two levels each, whereas the factor response sequence codes the three levels of all possible target and response sequences (i.e., target repetition with response repetition, target switch with response repetition, and target switch with response switch). ANOVA effects for which the sphericity assumption was violated were corrected by the Greenhouse-Geisser method, and we provide the corresponding ɛ estimate for each of these tests.

DV	SOURCE	F	P	η_P_^2^	ɛ

RT	Distractor Type (DT)	3.59	.065	0.08	

	Distractor Sequence (DS)	24.49	<.001	0.38	

	Response Sequence (RS)	131.84	<.001	0.77	

	DT * DS	1.13	.294	0.03	

	DT * RS	1.53	.223	0.04	

	DS * RS	36.61	<.001	0.48	

	DT * DS * RS	0.00	.998	0.00	

PE	Distractor Type (DT)	1.34	.254	0.03	

	Distractor Sequence (DS)	6.23	.017	0.13	

	Response Sequence (RS)	58.77	<.001	0.60	

	DT * DS	0.57	.455	0.01	

	DT * RS	0.05	.915	0.00	.757

	DS * RS	27.01	<.001	0.40	

	DT * DS * RS	0.87	.401	0.02	.790

|ΔRD|	Distractor Type (DT)	0.02	.885	0.00	

	Distractor Sequence (DS)	0.45	.507	0.01	

	Response Sequence (RS)	57.47	<.001	0.59	.573

	DT * DS	1.99	.166	0.05	

	DT * RS	0.40	.671	0.01	

	DS * RS	3.30	.042	0.08	

	DT * DS * RS	0.14	.867	0.00	

**Table A6:** Binding and retrieval effects for response times (RT [ms]), percentages of commission errors (PE [%]), and absolute differences in response durations across successive trials (|ΔRD| [ms]) for Experiment 2. Separate binding and retrieval effects were computed for targets and responses (T-R) as well as for distractors and responses, with the latter being computed on the entire dataset first (D-R) and also on a reduced dataset after excluding target repetition trials (D-R’).

		DIRECT OFFSET			DELAYED OFFSET		

DV	STATISTIC	T-R	D-R	D-R’	T-R	D-R	D-R’

RT	Mean	63.03	29.57	21.76	78.46	29.76	22.14

	SE_M_	4.76	5.69	6.57	5.07	4.48	6.31

	d_z_	2.07	0.81	0.52	2.42	1.04	0.55

PE	Mean	6.75	3.76	3.99	7.08	3.26	2.70

	SE_M_	0.75	0.60	0.96	0.69	0.75	0.95

	d_z_	1.40	0.98	0.65	1.61	0.68	0.44

|ΔRD|	Mean	0.05	0.64	–0.05	1.26	0.23	–0.29

	SE_M_	0.37	0.62	0.71	0.41	0.66	0.80

	d_z_	0.02	0.16	–0.01	0.48	0.06	–0.06
